# Performance enhancement of the soft robotic segment for a trunk-like arm

**DOI:** 10.3389/frobt.2023.1210217

**Published:** 2023-07-17

**Authors:** Shaowu Tang, Kailuan Tang, Shijian Wu, Yin Xiao, Sicong Liu, Juan Yi, Zheng Wang

**Affiliations:** ^1^ Shenzhen Key Laboratory of Intelligent Robotics and Flexible Manufacturing Systems, Southern University of Science and Technology, Shenzhen, China; ^2^ The Department of Mechanical and Energy Engineering, Southern University of Science and Technology, Shenzhen, China; ^3^ School of Mechatronics Engineering, Harbin Institute of Technology, Harbin, China

**Keywords:** soft arm, origami, modular, workspace, lateral stiffness, bending moment

## Abstract

Trunk-like continuum robots have wide applications in manipulation and locomotion. In particular, trunk-like soft arms exhibit high dexterity and adaptability very similar to the creatures of the natural world. However, owing to the continuum and soft bodies, their performance in payload and spatial movements is limited. In this paper, we investigate the influence of key design parameters on robotic performance. It is verified that a larger workspace, lateral stiffness, payload, and bending moment could be achieved with adjustments to soft materials’ hardness, the height of module segments, and arrayed radius of actuators. Especially, a 55% increase in arrayed radius would enhance the lateral stiffness by 25% and a bending moment by 55%. An 80% increase in segment height would enlarge 112% of the elongation range and 70 % of the bending range. Around 200% and 150% increments in the segment’s lateral stiffness and payload forces, respectively, could be obtained by tuning the hardness of soft materials. These relations enable the design customization of trunk-like soft arms, in which this tapering structure ensures stability via the stocky base for an impact reduction of 50% compared to that of the tip and ensures dexterity of the long tip for a relatively larger bending range of over 400% compared to that of the base. The complete methodology of the design concept, analytical models, simulation, and experiments is developed to offer comprehensive guidelines for trunk-like soft robotic design and enable high performance in robotic manipulation.

## 1 Introduction

Elephant trunks and octopus tentacles exhibit amazing dexterity and robustness in hunting and feeding (Wilson et al., 1991; Richter et al., 2015; Wu et al., 2018). Inspired by such dexterous appendages found in natural-world creatures, numerous applications in robotics have been made for manipulation (Hannan and Walker, 2003; Calisti et al., 2011; Bao et al., 2020) and grasping (Wang et al., 2019; Xie et al., 2020). In particular, as a quickly emerging research field, soft robotics relies on inherent compliance and flexibility similar to biological features resulting from soft materials (Kim et al., 2013; Rus and Tolley., 2015; Laschi et al., 2016). With this unique feature, widely explored trunk-like soft continuum robots have demonstrated the superior performance of extending and bending movementswith an infinite number of degrees of freedom by continuously stacking multiple segments and have shown high adaptability to handle various complex environments (Chen et al., 2019; Guan et al., 2020; Chen et al., 2021; Zhao et al., 2021). This suggests that trunk-like soft arms have the potential for wide applications. However, the performance in workspace and payload is heavily restricted by their continuum configuration, leading to explorations ranging from soft actuators to integrated robotic systems.

Efforts to improve the performance through materials and structural designs of soft actuators have been pursued for the past few decades (Polygerinos et al., 2015a; Xie et al., 2020). For instance, as the core unit of robots, pneumatically driven soft actuators have utilized such reinforced structures as fibers and rigid skeletons to largely enhance the capabilities of spatial movements and output forces (De Volder et al., 2011; Polygerinos et al., 2015b; Wang et al., 2016; Paterno et al., 2018). Especially, recent developments in soft origami actuators have enabled improved programmable performance (Martinez et al., 2012; Yi et al., 2018a; Yi et al., 2018b; Su et al., 2020; Shen et al., 2021). Based on these fundamental research works, the developments of soft continuum robots have been facilitated by integrating these soft actuators in parallel and longitudinal directions (Bishop-Moser et al., 2012; Chen et al., 2019; Qiao et al., 2019; Chen et al., 2021; Li et al., 2022), presenting individually controllable degrees of freedom for controllable workspace extensibility benefiting from the coordination among soft actuator modules. More recently, soft-rigid hybrid robots with rigid constraints to partially reinforce the structural stiffness have demonstrated improved payload and workspace of soft continuum robots (Zhou et al., 2018; Su et al., 2020; Liu et al., 2022). These remarkable efforts have greatly enhanced the capabilities of soft robots and motivated related work in soft robotics. Alternatively, approaches to the arrangement of soft actuators also have an influence on the robotic performance (Robertson et al., 2017; Olson et al., 2019; Zhang et al., 2020; Brancadoro et al., 2020). It has been pioneeringly verified by considering McKibben actuators and other fiber-reinforced actuators. This approach is coincident with the biological features of trunks where the elephant’s trunk is constructed almost entirely of muscle with totally soft tapering structures (Wilson et al., 1991). The arrangement of muscles provides not only the necessary forces for movement but also the forces needed to support the trunk structure (Hannan and Walker, 2001), thus showing the potential of this mechanism for performance enhancement. This leads to more general analyses on these approaches to the desirable robotic performance.

In this paper, we propose a design rule of the soft origami modular (SOM) segment and reveal that its performance could be largely enhanced by tuning the key design parameters. With the developed theoretical analysis, finite-element method (FEM) simulation, and experimental validation, it is verified that a larger workspace, lateral stiffness, payload, and bending moment could be achieved with adjustments to soft materials’ hardness, the height of module segments, and arrayed radius of actuators. Especially, a 55.56% increase in the arrayed radius would enhance the lateral stiffness by 25.18% and a bending moment by 55.59%. An 80% increase in segment height would enlarge 112.66% of the elongation range and 70.84% of the bending range. Around 200% and 150% increments in the segment’s lateral stiffness and payload forces, respectively, could be obtained by tuning the hardness of soft materials. These relations enable the design customization of trunk-like soft arms, in which this tapering structure ensures stability by the stocky base for an impact reduction of 50% compared to that of the tip and ensures dexterity of the long tip for a relatively larger bending range of over 400% compared to that of the base.

The main contributions of this paper include the following: 1) The design rule of the trunk-like robot’s modular segment reveals the key design parameters (the soft materials’ hardness, height of module segment, and arrayed radius of actuators) to the important capabilities (workspace, lateral stiffness, axial force, and bending moment) for enhanced performance customization. 2) Whole methodology on the design and analytical, simulated, and experimental methods for verifying the principles and guiding the design. 3) Development of the trunk-like soft robotic arm guided by the design rule, showing enhanced stability in the fixed end segment and dexterity in the tip.

## 2 Concept, modeling, and design

**FIGURE 1:**
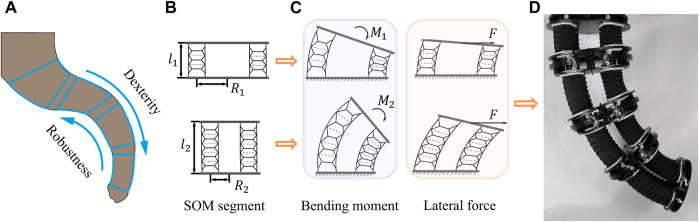
Concept of the SOM segment. **(A)** Dexterous and robust elephant trunk. Two SOM segments with different parameters **(B)** and exhibiting different performances **(C)**. **(D)** Trunk-like soft arm stacked by four segments.

Our core design rule concept for constructing a trunk-like soft robotic arm has two aspects to consider. On one hand, the important capabilities of the trunk-like robot’s module segment depend entirely on its design parameters; on the other hand, referring to the biological characteristics of elephant trunks, module segments in different positions (from end to tip) have different capabilities and requirements such as the stability and robustness of the fixed end and dexterity of the free tip (see Figure 1). With this prerequisite, the desired performance enhancement SOM segments are enabled by tuning the arrayed radius, height, and hardness of soft materials, which will be discussed in the following modeling.

Modeling of the SOM segments starts from characteristics of origami actuators. Based on our previous work on various origami actuator designs (Guo et al., 2019; Liu S et al., 2021), the soft origami actuator with axial extension/contraction motions and omnidirectional passive compliance is utilized in this study.

**FIGURE 2:**
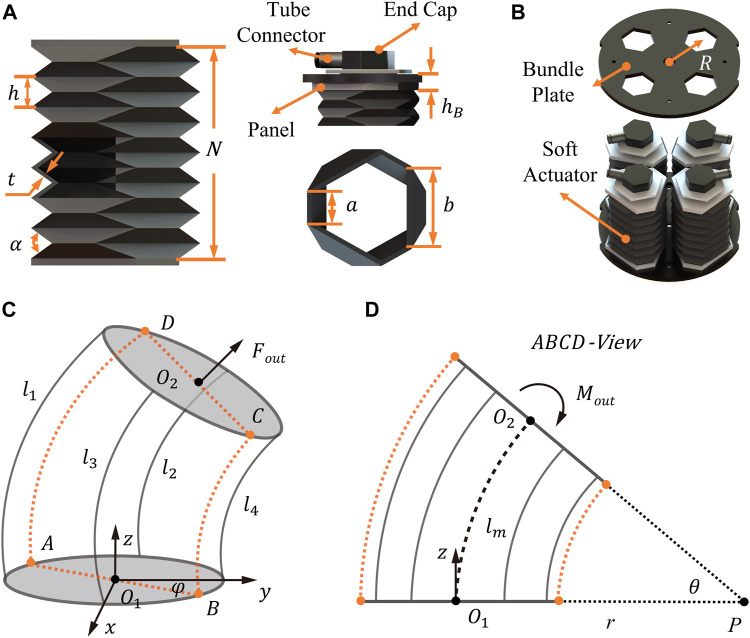
Modeling and design of the SOM segment. **(A, B)** Design drawings of the origami actuator and SOM segment. **(C, D)** Schematic sketches of a bending segment from a 3D view and ABCD plan view.

The soft origami layer is as shown in Figure 2A; its basic configuration is a hexagon with unequal long and short sides. The total length $l_{0}$ and cross-sectional area $S_{e}$ of the soft origami part can be expressed as

$$l_{0}=h_{0}N,$$ (1)

$$S_{e}=\frac{\sqrt{3}}{4}\left(a^{2}+b^{2}+4ab\right),$$ (2)

where $h_{0}$ is the initial height of one origami layer, N is the number of layers, and a and b are the side lengths of the hexagon. The origami dihedral angles can be compressed or expanded by supplying the corresponding pressure to the actuator’s chamber, causing the actuator to elongate or contract in the axial direction. Here, when the actuator elongates freely without hindrance, its total length l can be expressed as

$$l=l_{0}+2h_{B}+\Delta l,$$ (3)

where Δl is the elongation of the actuator and $h_{B}$ is the thickness of the panel. On the other hand, when the actuator’s elongation is hindered, the axial payload force f to outside can be expressed as

$$f=pS_{e}-K\cdot \Delta l,$$ (4)

where p is the internal pressure and K is the stiffness coefficient of the soft origami actuator, which is related to the hardness of the soft material.

Soft actuators exhibit high non-linearity owing to the elastic materials and flexible structure designs, so there is also a non-linear relationship between the actuator’s elongation and pressure. This non-linearity had been ignored in our previous modeling work (Guo et al., 2019; Liu J et al., 2021; Shen et al., 2021), resulting in a relatively large error between the fitted curve and the actual performance. Therefore, a quadratic function is used to fit the relationship between the pressures and displacements in following modeling:

$$\Delta l=k_{1}p^{2}+k_{2}p+k_{3},$$ (5)

where $k_{1},k_{2},k_{3}$ are the polynomial coefficients which will be fitted in the validation section. So far, we have modeled the elongation and axial payload force of a single soft origami actuator. Then, we will model the performance of a module segment composed of four parallel actuators.

By parallelly bonding four origami actuators into the two same plates (see Figure 2B), duo to the compliance, the SOM segment is capable of the spatial linear movements and omnidirectional-bending movements. As a result, the related workspace, axial payload force, and bending moment are modeled. Figures 2C,D show the spatial postures of the segment in a Cartesian coordinate system.

We assume that when the internal pressure of each actuator changes, ignoring the antagonism between actuators, these four actuators will undergo different deformations and eventually leave the segment in a steady state with constant curvature (Camarillo et al., 2008), thus causing the segment to bend with a bending angle θ , equivalent height $l_{m}$ , and a drift angle φ with the constant curvature radius r as shown in Figures 2C,D. These four posture variables $l_{m}$ , θ , r , and φ are directly related to the four actuators’ length and their arrayed radius. Their length is defined as $l_{i}$ ( i=1,2,3,4 ) and arrayed radius as R ; then, their relationship can be expressed as

$$\left\{\begin{array}{l} l_{1}=\theta \left(r+R\;\cos\;\varphi \right)\\ l_{2}=\theta \left(r+R\;\sin\;\varphi \right)\\ l_{3}=\theta \left(r-R\;\sin\;\varphi \right)\\ l_{4}=\theta \left(r-R\;\cos\;\varphi \right) \end{array}\right..$$ (6)

The system of Eq 6 is solved, and these posture variables $l_{m}$ , θ , φ , and r can be expressed as

$$l_{m}=\frac{l_{1}+l_{3}}{2}=\frac{l_{2}+l_{4}}{2}=\frac{1}{4}\sum_{i=1}^{i=4}l_{i},$$ (7)

$$\theta =\frac{\sqrt{{\left(l_{1}-l_{4}\right)}^{2}+{\left(l_{2}-l_{3}\right)}^{2}}}{2R},$$ (8)

$$\varphi =\tan{\left(\frac{l_{2}-l_{3}}{l_{1}-l_{4}}\right)}^{-1},$$ (9)

$$r=\frac{2\left(l_{1}+l_{4}\right)R}{\sqrt{{\left(l_{1}-l_{4}\right)}^{2}+{\left(l_{2}-l_{3}\right)}^{2}}}.$$ (10)

Equations 7-10 reveal that the posture of the segment can be fully defined by lengths of the four actuators and their arrayed radius. Based on the posture variables, the position of the upper plate’s center point $O_{2}$ in the Cartesian coordinate system can be further calculated as

$$\left\{\begin{array}{l} O_{2-x}=r\left(1-\cos\theta \right)\;\sin\varphi \\ O_{2-y}=r\left(1-\cos\theta \right)\;\cos\;\varphi .\\ O_{2-z}=r\;\sin\theta \end{array}\right.$$ (11)

So far, Eqs 6–11 establish the position of a single segment’s endpoint in Cartesian coordinates and the parameters affecting the segment workspace have been determined. Then, the payload performance including axial force and bending moment will be modeled. Axial payload force $F_{out}$ and bending moment $M_{out}$ can be obtained by summing the output force of each actuator as follows:

$$F_{out}=\sum_{i=1}^{i=4}f_{i},$$ (12)

$$M_{out}=\sum_{i=1}^{i=4}f_{i}\cdot R_{i},$$ (13)

where $R_{i}$ is the coefficient moment arm, which can be expressed as

$$\left[\begin{array}{cccc} R_{1} & \;R_{2} & R_{3} & R_{4} \end{array}\right]=R\;\cdot \left[\begin{array}{cccc} \cos\varphi & \sin\;\varphi & -\sin\;\varphi & -\cos\;\varphi \end{array}\right].$$ (14)

Equations 12 and 13 elaborate the important performance of axial payload force and bending moment are both related to the axial output force of actuators.

Substituting Eq 4 in Eqs 12, 13, we can calculate the relationship between the SOM segment’s payload force, bending moment, and these actuators’ internal pressure and elongation as

$$F_{out}=\sum_{i=1}^{i=4}\left(p_{i}S_{e}-\right.\left.K|\cdot |\Delta |l_{i}\right),$$ (15)

$$M_{out}=\sum_{i=1}^{i=4}\left[\left(p_{i}S_{e}-K\cdot \Delta l_{i}\right)R_{i}\right].$$ (16)

As a result, Eqs (7)–(11), 14, 15 are used to represent the workspace, axial payload force, and bending moment in this research, where the key parameters of arrayed radius, actuator dimensions, and soft material hardness are addressed. The relations will be used to guide the design and verified with simulations and experiments.

## 3 Fabrication and the actuation system

Following the models, six SOM segment variants are designed, which are named " R - N -Shore Hardness.” R is the radius of the actuator array, N is the number of origami layers (positively correlated with segment height), and Shore hardness is that of soft materials (Meththananda et al., 2009). Details are listed in Table 1. Other parameters of the origami actuators are given in Table 2.

**TABLE 1:** Name and parameters of the six SOM segment variants.

Name	R (mm)	N	Shore hardness (A)
36-7-90A	36	7	90
36-5-90A	36	5	90
36-9-90A	36	9	90
46-7-90A	46	7	90
56-7-90A	56	7	90
36-7-70A	36	7	70

**TABLE 2:** Geometry of the soft origami actuator.

Symbol	Parameter	Dimension
$h_{0}$	Height of one origami layer	8.6 mm
$h_{B}$	Height of the bundle plate	7.8 mm
t	Thickness of origami facets	1.1 mm
a	Length of the short side of the hexagon	13.55 mm
b	Length of the long side of the hexagon	33.55 mm
α	Dihedral angle of two trapezoid facets	73.4°

To explore the influence of parameters on module segment performance, these six variants in Table 1 are fabricated. First, to endow segments with an infinite number of degrees of freedom and omnidirectional movement, the soft materials selected for origami actuators are a type of polyurethane-based silicone rubber Hei-Cast 8400, and we can flexibly adjust the Shore hardness by changing the proportion of Hei-Cast 8400 liquids A, B, and C (Wang et al., 2021).

**FIGURE 3:**
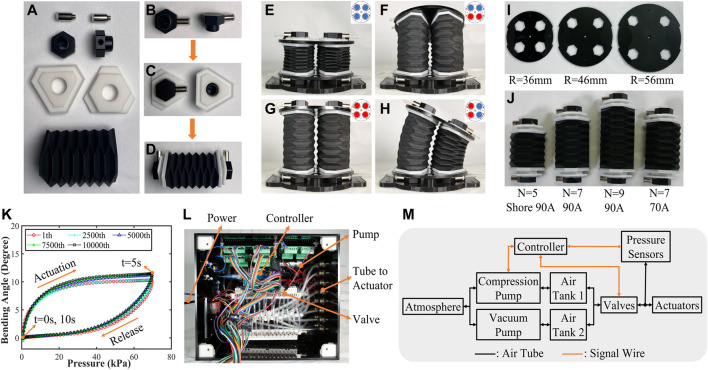
**(A)–(D)** Fabrication process of the soft origami actuator. **(E)–(H)** Three-DOF segments and corresponding actuation schemes are recorded. Three groups of bundle plates with different arrayed radius **(I)** and three groups of the number of origami layers and two groups of Shore hardness **(J)** are fabricated. **(K)** The fatigue test repeated 10,000 times. **(L)** Actuation system box, with controller, pumps, and valves to actuate the segments. The dimensions of the box are 31 cm*26 cm*20 cm. **(M)** Control system sketch for pneumatic actuators.

Then, two tube connectors (KQ2S04-M5, SMC Co.), two end caps (made of aluminum alloy, CNC machining), two panels (made of nylon, 3D printing), and one soft origami part are used to fabricate one soft origami actuator as shown in Figure 3A, and the steps are as follows: 1) The connections are assembled to the end caps by M5 threads, and the extruded rubbers provide the airtightness of this threaded assembly as shown in Figure 3B. (2) The hole diameter of the panel is 16 mm, which is larger than the shaft diameter of the end cap (15 mm); the methyl acrylate glue is filled in the gap between the hole and shaft to play a role of bonding and sealing as shown in Figure 3C. (3) The end faces of the soft origami part are set in the hexagonal groove of panels (width 4.5 mm and depth 1.8 mm), and they are bonded and sealed by cyanoacrylate adhesive. The overall appearance of the soft origami actuator can be seen in Figure 3D. After completing the fabrication of the actuator, the assembly of the SOM segment is carried out. Two bundle plates and four actuators are assembled into an SOM segment with three DOFs as shown in Figures 3E–H.

Based on the conclusions from the previous analysis, the Shore hardness, height of module segments, and arrayed radius of actuators are the three principal parameters affecting the performance of the SOM segment. According to these parameters, three kinds of radius bundle plates and four kinds of actuators were prepared as shown in Figures 3I,J.

So far, six variants have been fabricated. To ensure that these variants are workable and can be actuated repeatedly, the 36-7-90A segment is used as an example to perform the 10,000 repetitive fatigue tests, and the results are plotted in Figure 3K. In this test, 0 kPa–70 kPa air pressure was continuously given into one actuator, resulting in a bending cycle within 10 s. The results demonstrate that the module is able to work properly after the 10,000 cycles (potentially an even higher number of cycles). Figure 3K shows that the joint module works in consistence with RMSE of 0.2° in the 5,000th and the 10,000th bending cycles.

For uniform and efficient experimentation, an actuation system was developed as shown in Figures 3L,M, which mainly consists of one controller board (Arduino Mega 2560), two air pumps (KVP8 PLUS- KB-S, Kamer), two air tanks, and 32 pneumatic solenoid valves (T103U-BM, OST). To improve the ability of actuation and experimentation, open-loop control is used in this system. In detail, the pressure inside the actuators is indicated by a pressure sensor (ISE30A-01-N, SMC). The pressure values are relatively stable due to the good airtightness and responsiveness of solenoid valves. The user then sends corresponding commands to the controller by observing the pressure sensors to increase or decrease the internal pressure values.

## 4 Simulation and experimental validation

### 4.1 Finite-element method simulation

In addition to the design parameters, the hyperelasticity of the material and the antagonism between the actuators also affect the module segment performance which is ignored in analytical models. Therefore, it is necessary to establish a more accurate and comprehensive simulation model to reflect the module segment’s omnidirectional motion and performance. In this work, the finite-element simulations are conducted in ABAQUS/CAE (Dassault Systemes). Here, boundary conditions and preprocessing models are elaborated by taking the simplified SOM segment 36-7-70A as an example.

**FIGURE 4:**
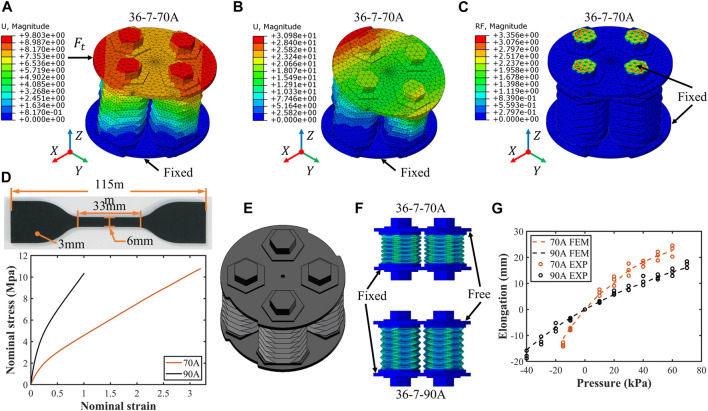
Setup and validation of the finite-element simulation model. **(A)–(C)** Visualization of the different simulations and the corresponding boundary conditions in the software ABAQUS/CAE. **(D)** Dumbbell-shaped specimens made of Hei-Cast 8400 with Shore hardness 70A and 90A and the nominal strain–stress curve for uniaxial tensile strength test data. **(E)** Simplified segment assembly for finite-element simulations to reduce computational effort. A simulation test of segment elongation **(F)** is used to validate the rationality of this finite-element model, and results are shown **(G)**.

For the boundary conditions, the three simulations on lateral stiffness, workspace, and bending moment are configured separately. The lateral stiffness boundary conditions are as shown in Figure 4A; the base plate is fixed, and the lateral force is applied to the upper bundle plate. The displacement of the center node of the upper plate is obtained in the visualization interface. Then, the lateral stiffness can be obtained by a simple calculation of dividing the force by the displacement. In the SOM segment’s workspace simulations (see Figure 4B), the base plate is also fixed, and the pressure is applied to all the inner walls of the origami actuators. By using the probe to obtain the displacement of the specified node on the upper plate, we can calculate the position and orientation of segments. In payload force and bending moment simulations, the base plate and four specific surfaces of the upper plate are fixed as shown in Figure 4C. According to Newton’s third law of motion action and reaction are equal and opposite, we can calculate the payload force and bending moment by summing these nodes’ reaction force in the four fixed surfaces. So far, we set the simulation boundary conditions for all the tests. Then, the preprocessing of these simulation tests will be carried out.

For preprocessing, we mainly consider the property of soft materials. To ensure that the simulated and real data are uniform, multiple Shore hardness 70A and 90A dumbbell-shaped specimens (see Figure 4D) are tested for uniaxial tensile strength according to a computer-controlled universal testing machine (MTS Criterion Model 42) at a rate of 200 mm/min. The obtained nominal stress-nominal strain curve before fracture of the specimens is shown in Figure 4D. Due to the hyperelastic and incompressible properties, the Mooney–Rivlin model, with strain energy density function $C_{ij}=\delta_{ij}\lambda_{i}^{-2}$ , is used to describe the non-linear behavior of this material (Rivlin, 1948). In particular, the material coefficients were $C_{10}=1.108$ MPa, $C_{01}=0.555$ MPa (Shore hardness 70A) and $C_{10}=-0.436$ MPa, $C_{01}=5.949$ MPa (Shore hardness 90A). On the other hand, the rigid aluminum plate is considered a linearly elastic material with a Young’s modulus of 70 MPa and a Poisson’s ratio of 0.3.

After setting up the conditions and parameters, the correctness and accuracy of these simulations should be tested. A group of elongation simulation tests are carried out as shown in Figure 4F. The base plate is fixed while the upper plate is free. When equal pressure is applied to the chamber of the four origami actuators, the segment elongates. The results (see Figure 4G) show that the simulation model is stable and effective in capturing the behavior of the SOM segment. Therefore, both the analytical model and the simulation model are used in the analysis of the following experimental results.

### 4.2 Workspace tests

**FIGURE 5:**
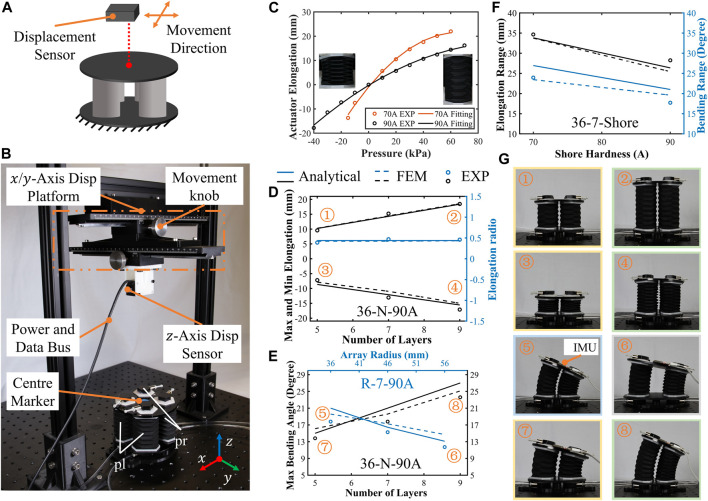
Tests of the SOM segment workspace. **(A)** Sketch of the test setup, with a fixed base plate and free upper plate and a movable laser displacement sensor for tracking the center marker of the upper plate. **(B)** Test setup; the displacement center marker in three axes and the internal pressure of the four actuators are recorded. **(C)** Experimental results and curve fitting of the origami actuator’s elongation. **(D)** Module segment elongation test results with internal pressure 60 kPa and −30kPa; the elongation against the number of origami layers is plotted. **(E)** Results of the maximum bending angle against the number of origami layers and actuator array radius. **(F)** Results of the elongation range and bending range against Shore hardness. **(G)** Design parameters have a significant influence on the working range.

Model (7)–(11) established the relationship between the workspace of SOM segments and actuators’ elongation $\Delta l_{i}$ , length $l_{0}$ , and arrayed radius R . Among these three parameters, the length and arrayed radius are known for a specific segment, and the elongation varies with the internal pressure of the actuator as in Eq 5. Here, we further consider the relationship between elongation and internal pressure by the curve fitting method through experiments as shown in Figure 5C where the experimental procedure is quasi-static and the hysteresis of the soft material is ignored. From the experimental results, the minimum and maximum pressure supplied to the 70A actuator are −15 kPa and 60kPa, and those supplied to the 90A actuator are −40kPa and 70 kPa. Due to the non-linearity between elongation and pressure, the elongation rate of these two actuators gradually decreases with an increase in pressure; the quadratic function as shown in Eq 5 is used to fit curves of elongation against internal pressure with 70A and 90A, and the fitted coefficients and quadratic functions are as follows:

$$\Delta l_{i}^{7-70A}=-0.0052p_{i}^{2}+0.6857p_{i}-0.9435,$$ (17)

$$\Delta l_{i}^{7-90A}=-0.0017p_{i}^{2}+0.3444p_{i}-0.2562,$$ (18)

where $\Delta l_{i}^{7-70A}$ and $\Delta l_{i}^{7-90A}$ are the elongation of the origami actuator with seven origami layers and $p_{i}$ is the internal pressure. Since the pressure on all inner walls of the actuator is the same, for actuators with a different number of layers, their elongation needs to be multiplied by the layer’s ratio on model (17)–(18). After substituting model (17) and (18) into model (7)–(11), the relationship between the SOM segment’s workspace and the actuator’s internal pressure has been established. This analytical model will be used to discuss the results in the following tests.

Previously, the relationship between workspace and pressure is established by data fitting. Here, we focus on validating the relationship between the segment’s workspace and the material’s hardness, height, and arrayed radius. The test setup is shown in Figures 5A,B. A high-accuracy laser displacement sensor (HG-C1200, Panasonic) is fixed to a displacement platform with a scale in the x - and y -direction. The base plate is fixed, and the center of the upper plate is marked with a point. When the position of the upper plate changes, the laser of the sensor is used to track the marker point by turning the movement knob and record the displacement of this sensor to the marker point. In this test setup, the position of the upper plate center is recorded.

When the pressures inside the four actuators are equal, these actuator’s deformations are synchronized, the elongation tests are carried out, and the results are plotted in Figure 5D. Since the value of pressure is an irrelevant variable in these tests, to reduce damage and loss, the actuators are not supplied with extreme pressure. In these tests, the maximum elongation (internal pressure is 60 kPa) and the minimum elongation (internal pressure is −30 kPa) of the three SOM segments with different origami layers were measured. The 36-5-90A segment’s elongation range is 16.75 mm which equals to maximum elongation 9.55 mm (60 kPa) minus minimum elongation −7.2 mm (−30 kPa); 36-7-90A and 36-9-90A segments’ elongation is 28.25 and 35.52, respectively. The results show that both simulation and analysis methods can capture the characteristics of the segment’s elongation, and the RMSE between the analytical and experimental range is 1.82 mm, verifying the accuracy of models in elongation predictions. On the other hand, experimental results show that the elongation ratio (elongation range divided by original length) remains almost unchanged at about 50%. It suggests that the elongation range is exactly proportional to the number of layers, which is about 3.75 mm/layer.

When the internal pressures of the four actuators are unequal, the segments will bend in the corresponding direction due to the constraints of the bottom and upper plate, and then, the bending range tests are carried out. In these tests, we explored the bending range of segments under the same actuation pressure in the x – z plane. The internal pressures of the two actuators in the positive direction of the *x*-axis (pl in Figure 5B) are 60 kPa, and the internal pressure of the other two (pr) are −30 kPa. The bending range data as shown in Figure 5E are measured and recorded by the inertial measurement unit (IMU, CH100). The results demonstrated that both the number of origami layers and the actuator array radius influence the maximum bending range. Among these segments, the one with the largest bending range (23.6°) is the 36-9-90A segment and the smallest (11.7°) is the 56-7-90A segment. In addition, the maximum error between the experimental results and the simulation results is 3° and between the experimental results and the analytical results is 3.4°. The RMSE between the total analytical and experimental bending ranges is 2.24°. These bending errors might be due to complex antagonism interactions between the four actuators. The main reason for the deviation between the experimental results and analytical models is the antagonism among soft actuators. For a module segment with parallelly arranged actuators, movements of one actuator could be transmitted to other actuators through the rigid panel, forming an antagonism. It is ignored in our model in this work, resulting in experimental results that are always lower than the analytical prediction.

So far, we tested and analyzed the relationship between the design structural parameters and the segment’s workspace. For a soft module segment or soft robotic arm, the material’s hardness is also an important parameter that affects the workspace. The comparison of the workspace of the 36-7-70A segment and 36-9-90A segment is shown in Figure 5F. From the results, both the elongation range and bending range decrease with an increase in Shore hardness. The elongation range of the 36-7-90A segment is 28.25 mm under pressure between −30 kPa and 60 kPa, which is 18.49% less than the 34.66 mm of the 36-7-70A segment under pressure between −15 kPa and 60 kPa. The bending range of the 36-7-90A segment is 17.72° with pressure pl 60 kPa and pr −30 kPa, which is 26.01% less than the 23.95° of the 36-7-70A segment with pressure pl 60 kPa and pr −15 kPa. Among these results, the largest error is 15.66% in the bending range between the model result and test result of the 36-7-90A segment.

### 4.3 Lateral stiffness tests

**FIGURE 6:**
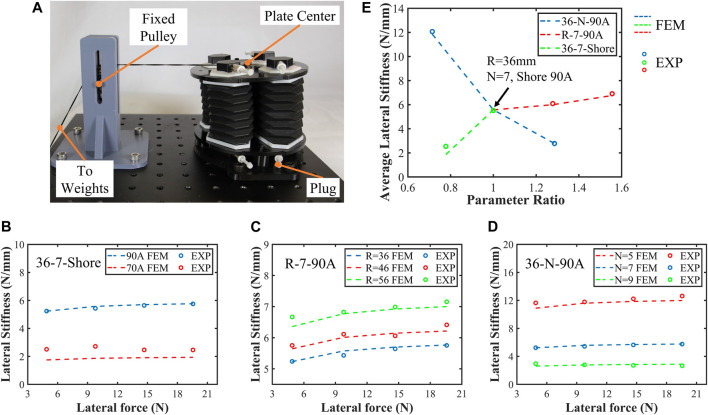
Tests of segment lateral stiffness. **(A)** Lateral stiffness test setup; the lateral force is provided by the weights where the gravitational acceleration is 9.78 m per second squared; the lateral displacement of the upper plate center was measured using a laser sensor as shown in Figure 5B. **(B)**–**(D)** Lateral stiffness results and three-set comparison of univariate variables. **(E)** Average lateral stiffness of the six SOM segments was plotted, where the horizontal coordinate is the normalized parameter ratio.

Stiffness is a measure of the resistance to deformation, and SOM’s lateral stiffness represents the performance of resisting lateral displacement when subjected to lateral force (Brancadoro et al., 2020; Liu et al., 2022). Its value is equal to the lateral force applied to the structure divided by the lateral displacement, which is related to the material properties and SOM segment structure. In this section, the relationships between lateral stiffness and the soft materials’ hardness, height of module segments, and arrayed radius of actuators are explored. The experimental setup for measuring lateral stiffness is shown in Figure 6A. The base plate of the segment is fixed. A fixed pulley and a non-stretchable rope were used to convert the gravity of the weights into a lateral force applied to the center of the upper plate. In addition, the platform in Figure 5B is also used in these tests to track the lateral displacement of the upper plate center. Throughout the experiments, the tube connectors of each actuator are plugged to keep the actuator chamber closed. To reduce the influence of material fatigue on the experimental results, there is an interval of 2 minutes between each two sets of experiments to allow segments to return to the initial state. In this setup, the gravity of weights, lateral displacement of the upper plates, and center of six SOM segments are recorded.

The results of lateral stiffness tests are plotted in Figures 6B–D where six SOM segments are grouped into three univariate variables for comparison. The test results show that the finite-element model can well describe the lateral stiffness characteristics of segments with Shore hardness 90A, with a maximum error of 4.6%. As for the 36-7-70A segment, there is a larger error due to greater elasticity and non-linearity. In addition to arrayed radius, Shore hardness, and actuator length, for these SOM segments, their lateral stiffness is also affected by lateral force. For example, for the 36-7-90A segment, when the lateral force is 19.6 N, the lateral stiffness is 0.575 N/mm, which is 0.051 N/mm larger than that when the lateral force is 4.9 N.

So far, we separately tested the relationship between the segment’s lateral stiffness and soft material hardness, height of the segment, and arrayed radius of the actuator. To compare the influence of different design parameters on the lateral stiffness, the average lateral stiffness of these six segments is plotted in Figure 6E where the horizontal coordinate is the normalized parameter ratio. It was shown that the average lateral stiffness is most influenced by the soft material hardness, where the average lateral stiffness of the 36-7-90A segment is 5.52 N/mm, 2.98 N/mm larger than that of the 36-7-70A segment (2.54 N/mm). This enhancement reaches 217.32%. In addition, among the six module segments, the lateral stiffness of the 36-5-90A segment is the largest at 6 N/mm and is 4.34 times that of the 36-9-90A segment. These results will be a critical guideline for the custom design of module segments and robotic arms.

### 4.4 Axial force and bending moment tests

**FIGURE 7:**
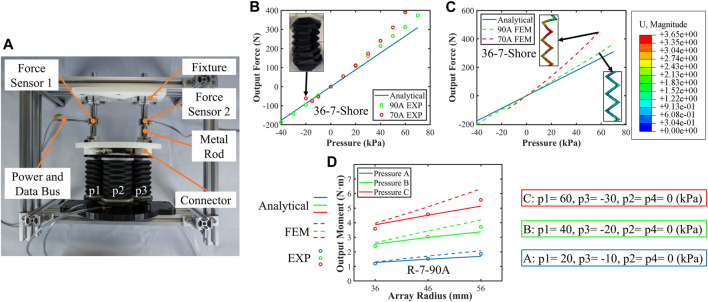
Tests of segment axial force and bending moment. **(A)** Test setup; the base plate and the upper plate were fixed; the output force and moment were transmitted to two force sensors. **(B)** Analytical and experimental results; two segments’ output force against internal pressure was plotted. **(C)** Analytical and FEM output force results. **(D)** Three segments of R-7-90A segment output moment results with the three groups’ pressure.

The payload capabilities of SOM segments are validated in this section. According to models (15) and (16), when two plates of the SOM segment are fully fixed, both axial payload force and bending moment depend on the output force of origami actuators. Following this principle, the segment is fixed in the experimental platform as shown in Figure 7A. In this platform, two axial force sensors (AR-DN102-500N, ARIZON) are mounted at the same distance to the upper plate center of 45 mm. Both force sensors are firmly mounted between the aluminum frame and the upper plate. With this setup, the axial payload force can be directly recorded by two force sensors while the bending moment can be calculated according to simple loading analysis. Two conditions are conducted to measure the axial force and bending moment. When four actuators are actuated with the equal values of inner pressure, axial payload forces of SOM segment will be measured. When four actuators are actuated with unequal pressure, bending moments are generated and recorded.

Model (15) indicates that the output force is independent of the arrayed radius and the number of origami layers. Here, two SOM segments of 36-7-70A and 36-7-90A are tested with experimental and analytical results plotted in Figures 7B,C. When the internal pressure value is positive, the axial force characteristics of these two segments are close, and the maximum output force of the 36-7-70A segment is 390.1 N, while that of the 36-7-90A segment is 373.2 N. When the internal pressure value is negative, the soft origami structure will be collapsed by the excessive pressure difference (see Figure 7B), causing the minimum axial force of the 36-7-70A segment to be −75.1 N while that of the 36-7-90A segment is −186.3 N. Almost 150% increments in the axial force can be achieved by replacing soft material from 70A hardness to 90A hardness, verifying the proposed design principle for performance enhancement. The same increasing tendencies are shown in the analytical and experimental results. Slight deviations could be observed due to the non-linear inflation of soft materials which was ignored in the analytical models.

In bending moment tests, three sets of pressure groups A, B, and C are supplied to four actuators and four pressure values in each group are not equal, where p1 is positive, p3 is negative, and p2 and p4 are zero. Test results of three segments of R-7-90A are measured and plotted in Figure 7D. Both the simulation and experimental results show that the maximum bending moments reaching to around 6 Nm with a pressure differential of 90 kPa meet the performance requirements of the trunk-like robotic arm at the base segment.

So far, we explored the relationships between the segment’s performance and design parameters by analytical, simulated, and experimental methods. The design rule of the SOM segment proposed that a larger workspace, lateral stiffness, and payload could be achieved with adjustments to the soft materials’ hardness, height, and radius of segments. To verify this, six SOM segments with different design parameters were fabricated and tested. Their performance (elongation range, bending angle, lateral stiffness, axial force, and bending moment) is listed in Table 3.

**TABLE 3:** Summarization of SOM segment performance.

Parameter	SOM	Elongation range (mm)	Bending range (degree)	Lateral stiffness (N/mm)	Axial force (N)	Bending moment (Nm)
Number of origami layers (36-N-90A)	36-5-90A	16.75	13.82	12.0	-	-
	36-7-90A*	28.25	17.80	5.52	-	-
	36-9-90A	35.62	23.61	2.78	-	-
Arrayed radius (R-7-70A)	36-7-90A*	-	17.80	5.52	-	3.58
	46-7-90A	-	15.27	6.09	-	4.57
	56-7-90A	-	11.72	6.91	-	5.57
Shore hardness (36-7-Shore)	36-7-70A	34.66	21.03	2.54	390.09	-
	36-7-90A*	28.25	17.80	5.52	373.16	-

36-7-90A* is the segment with benchmark design parameters. The radius, height, and hardness are adjusted based on this segment.

From the table, it could be found that there are always trade-offs between enlarging the workspace and increasing lateral stiffness. In particular, larger segment height, smaller segment radius, and softer materials would result in larger workspace while leading to smaller lateral stiffness. For instance, in this case, an 80% increase in segment height would enlarge 112.66% of the elongation range and 70.84% of the bending range while leading to limited lateral stiffness. A 55.56% increase in arrayed radius would enhance the lateral stiffness by 25.18% and a bending moment by 55.59% while resulting in a smaller workspace. These relation findings enable the performance customization of a trunk-like soft multi-segment arm.

### 4.5 Demonstration on the trunk-like soft robotic arm

**FIGURE 8:**
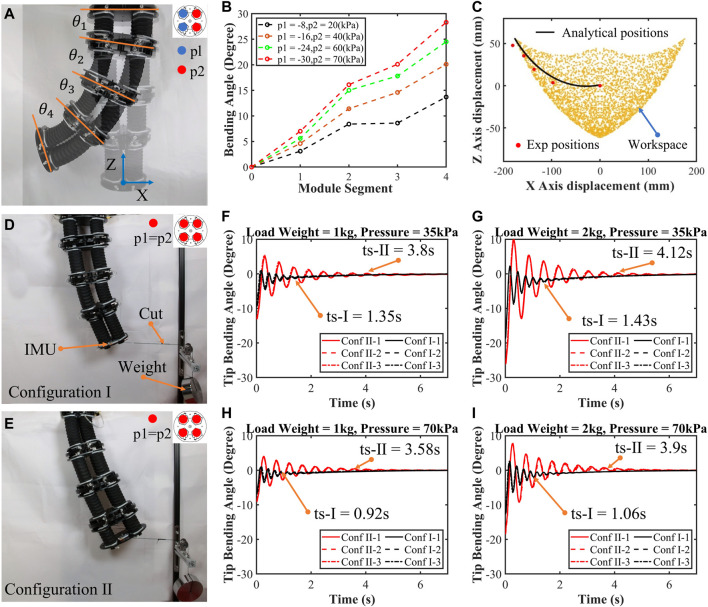
Demonstrations on the trunk-like soft robotic arm. **(A)** This arm was assembled from four segments: 66-5-90A, 56-7-90A, 46-7-90A, and 36-9-90A from the base to tip. **(B)** Bending test results are plotted **(B)**. **(C)**Analytical workspace and position tests of the arm’s endpoint. **(D–E)** Two configurations of the arm and the impact test results **(F–I)**.

Inspired by the biological characteristics of elephant trunks which have a stable and robust fixed end and dexterous free tip, the base segment of the trunk-like robotic arm should be thicker and shorter to ensure stability, while the tip is longer and thinner to ensure dexterity. Then, four SOM segments with specific performances (from base segment to tip: 66-5-90A, 56-7-90A, 46-7-90A, and 36-9-90A) are assembled into a robot arm as shown in Figure 8A. After the soft robotic arm is assembled, a bending test and an impact test are used to verify the enhancement of dexterity and robustness, respectively.

To verify the enhancement of dexterity, bending tests are conducted. The trunk-like robotic arm is hung vertically, and the strong base segment is fixed while the dexterous tip segment is free as shown in Figure 8A. The actuators in the same position as of four segments are ventilated by the air tube, so they have the same internal pressure. In addition, the data such as the segment’s angle θ and endpoint’s position in the Cartesian coordinate system are obtained from photos taken at a fixed camera position. Although this approach of recording data will add some reading errors, it has little effect on judgment of test results. In bending tests, vacuum is supplied to eight actuators on the left, while compressed air is supplied to the right eight actuators. The tests results show that the bending angle of each segment increases significantly from the base segment to the tip as shown in Figures 8A,B and Supplementary Video S1. The maximum tip segment bending angle is 28.3°, more than 400% times that of the base segment. The bending angle of the arm can be calculated by summing that of each segment, with a maximum of about 71.5°. On the other hand, the analytical workspace and trajectory in the X–Z plane (plotted from the positional iteration of Equations 1–11 of the arm’s endpoint and experiment result of the endpoint’s positions are shown in Figure 8C. From the initial state (p1 = p2 = 0 kPa) to the maximum bending state (p1 = −30kPa and p2 = 70 kPa), the agreement between the experimental and analytical position results demonstrates the arm’s good movement ability.

Like an elephant’s trunk, the thicker base segment provides better robustness to the arm. To verify the improvement of the arm’s robustness due to the tapered structure design, impact tests are performed on the two arms which have opposite configurations (from the base to tip segment, respectively, configuration Ⅰ: 66-5-90A, 56-7-90A, 46-7-90A, and 36-9-90A; configuration Ⅱ: 36-9-90A, 46-7-90A, 56-7-90A, and 66-5-90A) as shown in Figures 8D,E and Supplementary Video S2. The base segment is fixed, and the tip is loaded laterally by weights; the impact is created by cutting the rope. In these tests, the angle of the end face is recorded, and results are plotted in Figures 8F–I. The results show that, on one hand, with the increase in the actuation pressure and the decrease in the load, the impact amplitude and stabilization time are smaller and shorter, respectively. For example, in configuration Ⅰ, when the load weight is 2 kg and the actuation pressure is 35 kPa, the maximum amplitude is 5.44° and stabilization time is 1.43 s; when the load weight is 1 kg and the actuation pressure is 70 kPa, better robustness of the arm is exhibited with a maximum amplitude of 1.96° and stabilization time of 0.92 s. On the other hand, by comparing the experimental results, it could be easily found that the arm of configuration Ⅰ exhibits better robust performance compared with that of configuration Ⅱ under different conditions (reduction in stabilization time by 70% and amplitude by 50% in average). This validates that the performance of the soft arm can be enhanced and customized by adjusting the design parameters of the module segment.

Here, we tested motion and impact performance. These results enable the performance customization of the trunk-like soft arm (stacked by multiple segments with differential-parametric modular sets) where the base is thicker and shorter to ensure robustness, while the tip is longer and thinner to ensure dexterity. This approach offers comprehensive guidelines for soft robotic design.

To clearly state the performance enhancement of the customized module segment and arm designs, the main performance comparisons with other designs in the literature from the module segment to arm aspects are listed in Table 4. From this table, the performance-enhanced segment and arm demonstrate that a relatively lower actuation pressure (75 kPa) could induce a significantly high payload (390.1 N) and lateral stiffness (12.07 N/mm), while retaining a relatively large elongation range and bending angle.

**TABLE 4:** Comparison of the performance of segments and arms in the literature.

Comparison		Publications	This work	Zhou et al. (2022)	Liu et al. (2022)	Arleo et al. (2021)	Chen et al. (2019)	Liu et al. (2021)
Module segments	Performance	Lateral stiffness (N/mm)	12.07	∼2.33	-	-	-	∼6.82
		Axial force N)	390.1	-	108	-	196	0
		Elongation (mm)	35.62	48.3	7.25	48.08	133.3	0
	Basic parameters	Weight (g)	432.1	450	74.8	91.5	266.7	90.3
		Working pressure (kPa)	70	650	160	400	200	160
Arms	Performance	Bending angle (°)	71.5	-	60	137	100	62.4
	Basic parameters	Segments	4	3	4	2	3	4

## 5 Conclusion and future work

In this work, we revealed the design principle of the SOM segment for enhanced performance of the workspace, structural lateral stiffness, and payload, to explore the rule of customizing soft modular segments to facilitate trunk-like soft arms. To this end, the principal parameters behind the design and modeling of SOM segments were investigated, including the soft materials’ hardness, height of module segments, and arrayed radius of actuators. We built the relations of the performance and design parameters by analytically modeling the spatial kinematics and force of the segment. The FEM simulations and experimental validations were conducted to verify these important models. Following the proposed rule, by adjusting these parameters, the performance of the soft modular segment was enhanced. In particular, a 55.56% increase in arrayed radius would enhance the lateral stiffness by 25.18% and bending moment by 55.59%. An 80% increase in segment height would enlarge 112.66% of the elongation range and 70.84% of the bending range. Around 200% and 150% increments in the segment’s lateral stiffness and payload forces could be obtained by tuning the hardness of soft materials, respectively. To further evaluate the proposed methodology, a trunk-like soft robot stacked by four segments with differential-parametric modular sets was developed, its tapering structure ensures stability due to the stocky base for an impact reduction of 50% compared with that of the tip, and ensures dexterity of the long tip for a relatively larger bending range of over 400% more than that of the base. This whole methodology could be used as design guidance and provide the basis for high performance of the trunk-like soft arms.

In future work, more detailed structural parameters and performance of trunk-like robots will be further considered. The control system for kinematics and dynamic manipulation of the trunk-like robots will be developed. In addition, the gravitational effects, non-linear effects of soft materials, and antagonistic effects between the actuators will be tackled in analytical models.

## Data Availability

The original contributions presented in the study are included in the article/Supplementary Material; further inquiries can be directed to the corresponding authors.
